# Anti-HIV-1 Nanobody-IgG1 Constructs With Improved Neutralization Potency and the Ability to Mediate Fc Effector Functions

**DOI:** 10.3389/fimmu.2022.893648

**Published:** 2022-05-16

**Authors:** Angela I. Schriek, Marlies M. van Haaren, Meliawati Poniman, Gillian Dekkers, Arthur E. H. Bentlage, Marloes Grobben, Gestur Vidarsson, Rogier W. Sanders, Theo Verrips, Teunis B. H. Geijtenbeek, Raimond Heukers, Neeltje A. Kootstra, Steven W. de Taeye, Marit J. van Gils

**Affiliations:** ^1^ Department of Medical Microbiology, Amsterdam UMC, Amsterdam Institute for Infection and Immunity, University of Amsterdam, Amsterdam, Netherlands; ^2^ QVQ Holding BV, Utrecht, Netherlands; ^3^ Department of Experimental Immunohematology, Sanquin Research and Landsteiner Laboratory, Amsterdam UMC, University of Amsterdam, Amsterdam, Netherlands; ^4^ Department of Microbiology and Immunology, Weill Medical College of Cornell University, New York, NY, United States; ^5^ Department of Biology, Faculty of Sciences, Utrecht University, Utrecht, Netherlands; ^6^ VerLin BV, Utrecht, Netherlands; ^7^ Department of Experimental Immunology, Amsterdam UMC, Amsterdam Institute for Infection and Immunity, University of Amsterdam, Amsterdam, Netherlands

**Keywords:** HIV-1, nanobodies, neutralization, Fc fusion, Fc-mediated effector functions

## Abstract

The most effective treatment for HIV-1, antiretroviral therapy, suppresses viral replication and averts the disease from progression. Nonetheless, there is a need for alternative treatments as it requires daily administration with the possibility of side effects and occurrence of drug resistance. Broadly neutralizing antibodies or nanobodies targeting the HIV-1 envelope glycoprotein are explored as alternative treatment, since they mediate viral suppression and contribute to the elimination of virus-infected cells. Besides neutralization potency and breadth, Fc-mediated effector functions of bNAbs also contribute to the *in vivo* efficacy. In this study multivalent J3, 2E7 and 1F10 anti-HIV-1 broadly neutralizing nanobodies were generated to improve neutralization potency and IgG1 Fc fusion was utilized to gain Fc-mediated effector functions. Bivalent and trivalent nanobodies, coupled using long glycine-serine linkers, showed increased binding to the HIV-1 Env and enhanced neutralization potency compared to the monovalent variant. Fusion of an IgG1 Fc domain to J3 improved neutralization potency compared to the J3-bihead and restored Fc-mediated effector functions such as antibody-dependent cellular phagocytosis and trogocytosis, and natural killer cell activation. Due to their neutralization breadth and potency and their ability to induce effector functions these nanobody-IgG1 constructs may prove to be valuable towards alternative HIV-1 therapies.

## Introduction

A major obstacle for the eradication of human immunodeficiency virus type 1 (HIV-1) is the latent viral reservoir that persist in transcriptionally silent CD4^+^ infected T cells. Latent reservoirs are established during primary infection, and when transcriptionally latent are not recognized by the immune system, providing a life-long reservoir of replication-competent HIV-1 (1, 2). Antiretroviral therapy (ART) suppresses viral replication and prevents disease progression. However, ART does not eliminate the reservoir and viral rebound will occur after treatment interruption (3, 4). Consequently, these drugs require daily administration, with the possibility of drug resistance and adverse events. Therefore, alternative approaches for HIV-1 therapy or a functional cure are desirable (5, 6).

The discovery of broadly neutralizing antibodies (bnAbs) has been an important step forward in the search for alternative HIV-1 therapies. BnAbs recognize conserved regions of the HIV-1 envelope glycoprotein (Env) trimer, such as the CD4 binding site (CD4bs), V3-glycan, trimer apex or the membrane proximal external region (MPER) (7, 8) and exhibit exceptional potency and breadth, neutralizing more than 90% of circulating viral strains (9). Moreover, bnAbs can mediate killing of virus-infected cells, prevent cell to cell transmission and reduce plasma viral load and cell-associated viral RNA and DNA, which is believed to reflect the content of the viral reservoir (10–14).

A complementary approach for antiviral therapy is the use of the variable domain of heavy chain only antibodies, also known as nanobodies or VHH. These heavy chain only antibodies are naturally produced by the biological family *Camelidae* (15). Nanobodies against H5N1 hemagglutinin, severe acute respiratory syndrome coronavirus 2 (SARS-CoV-2), rabies virus and rotavirus are being investigated for therapeutic applications as they have shown to be super potent and broadly neutralizing molecules suitable for therapy (16–19).

An advantage of nanobodies for HIV-1 therapy is their relatively small size (15 kDa) which enables them to interact with the protein surface despite the high number of glycans on the HIV-1 Env that may not be accessible by immunoglobulin G (IgG). Nanobodies have a relatively concave shape and relatively long complementarity determining region 3 (CDR3) loop as compared to conventional variable domains, which allows recognition of otherwise cryptic epitopes (20). Experimental immunization of llamas yielded anti-HIV-1 nanobodies as potent as those from human elite controllers (21). The most potent anti-HIV-1 nanobody described is J3, targeting the CD4bs, which neutralized 96 of 100 strains tested (21). Two other nanobodies, 2E7 and 1F10, targeting the first heptad repeat (HR1) on gp41 and the V3 loop respectively, were able to neutralize some of the viral strains that were resistant to J3.

Through bioengineering, the binding affinity and potency of antibodies can be further improved, making them more applicable for therapeutic application. Firstly, bispecific or bivalent nanobodies can display enhanced affinities and potencies due to avidity binding. It was previously reported that a bivalent form of 2H10, a nanobody targeting the MPER, displayed an 20-fold increased affinity, hereby neutralizing various sensitive and resistant HIV-1 strains (22). Another bispecific nanobody consisting of J3 and 2E7 was found to enhance strain-specific neutralization (23). Secondly, fusion of anti-HIV-1 nanobodies to the Fc region (CH2-CH3) of IgG1 has been shown to extent half-life, enhance neutralization and increase cell-cell spread prevention (24). Similarly, a recent study showed that a SARS-CoV-2 nanobody IgG1 Fc fusion displayed enhanced affinity and increased neutralizing activity (25). An important advantage of a nanobody-IgG fusion is the possibility to kill infected cells via Fc-mediated effector functions such as antibody-dependent phagocytosis (ADCP) and antibody-dependent cellular cytotoxicity (ADCC) (26).

In this study multivalent versions of nanobodies J3, 2E7 and 1F10 were created to enhance binding to the HIV-1 Env. These constructs showed increased binding and enhanced neutralization potency. The nanobodies were additionally fused to a human IgG1 Fc domain which introduced the ability to mediate Fc-effector functions, and influenced neutralization capacity depending on the epitope and viral strain. Furthermore, bispecific antibodies were created, increasing neutralization breadth, making them attractive for therapeutic applications. These constructs may have the potential to be implemented as alternative HIV-1 therapies.

## Materials and Methods

### Cells

THP-1 cells were gifted by Karel van Dort from the department Laboratory for Viral Immune Pathogenesis at the AMC, and were cultured at 37°C, 5% CO_2_ in RPMI 1640 Medium (ThermoFisher) supplemented with 10% fetal calf serum (FCS, Gibco), 1/1000 Penicillin Streptomycin (P/S, Gibco). Cells were passaged 3 times a week to maintain a density of 0.5–1×10^6^ cells/mL. TZM-bl cells, a HeLa cell line, were obtained from the AIDS Reagent Program and were cultured at 37°C, 5% CO_2_ in DMEM (ThermoFisher) supplemented with 10% FCS, 1/1000 P/S. Cells were passaged twice a week to maintain a confluency of 10–90%. Human Embryonic Kidney (HEK)-293T cells (Invitrogen) were cultured in DMEM (ThermoFisher) supplemented with 10% FCS, 1/1000 P/S and passaged twice a week to maintain a confluency between 40–80%.

### Nanobodies

The monovalent nanobodies, 2E7, 1F10 and J3 and the bispecific constructs were produced and purified as described previously (27–30). In brief, nanobodies equipped with a C-terminal 6-His tag and either a FLAG- or Myc-tag were produced in *E.Coli* strain TG1 and purified via affinity chromatography using TALON Metal Affinity Resin (Takara Bio Inc.) The following bivalent constructs were either described previously or generated new by cloning codon optimized synthetic genes (Thermo Fischer Scientific) into the yeast production vector pYQVQ using SacI and Eco91I restriction sites: J3-15GS-2E7, J3-15GS-1F10, J3-20GS-J3, 2E7-35GS-2E7, 1F10-15GS-1F10 and J3 -20GS-J3-20GS-J3. This was transformed into S.cerevisiae strain VWK18 as described previously. The nanobodies were produced and purified by affinity chromatography using CaptureSelect resin as described before (31).

### Cloning

G-blocks were ordered in which the nanobody sequence was directly fused to the hinge and Fc domain of the IgG1 human heavy chain sequence. These DNA fragments (Integrated DNA Technologies, Inc) were designed with overhangs to facilitate cloning into the AbVec-HigG plasmid using the Gibson assembly technique, as described previously (32), using the restriction sites, XbaI and HindIII. In short: Gibson mix (BioLabs) was incubated with the vector and insert DNA for 1 hour at 50°C. After incubation, XL-1 blue cells were added to the Gibson mix, incubated on ice for 30 minutes before heat-shock activation (45 seconds, 42°C). The bacteria/plasmid mix was further incubated for 1 hour at 37°C with 2x Lysogeny broth (LB). The bacteria mixture was plated out on 2xLB plates containing 100 μg/ml ampicillin and incubated overnight at 37°C. After incubation, colonies were picked, grown and purified using the plasmid DNA purification kit (Machery-Nagel). The DNA was sequenced by Macrogen Europe B.V using Sanger sequencing and correct clones were selected for further use. Bispecific antibodies were created by introducing knob in hole mutations with the addition of a charge reversal strategy to minimize homodimerization. Using Q5 Site-Directed mutagenesis (BioLabs) the mutations: S354C, T366W, K409A or: Y349C, T366S, L368A, Y407V, F405K were incorporated into the corresponding antibody plasmid. Primers were designed using the NEBaseChanger tool (Biolabs). Q5 Hot Start High-Fidelity 1x master mix, 10 µM forward primer, 10 µM reverse primers and the template DNA (1–25 ng/µl) were mixed and exponentially amplified using PCR (25 cycles), replicating the plasmid DNA with the mutation. Subsequently the DNA was incubated with a mixture of Kinase, Ligase & DpnI (5 minutes RT) to allow phosphorylation, ligation and the degradation of template DNA. Transformation of XL-1 blue cells and validation of correct clones was done as described previously.

### Antibody Production and Purification

The nanobody-IgG1 constructs were transiently expressed in HEK-293F cells (Invitrogen). Cells were cultured in Freestyle medium (Life Technologies) at a density of 1×10^6^ cells/mL. At day 1, the cells were transfected using the antibody plasmid and 1 µg/µl PEImax (Polysciences) in a 3:1 ratio in OptiMEM. For the production of the bispecific antibodies, HEK-293F cells were transfected with the J3 IgG1-knob plasmid and either the 2E7-IgG1-hole or 1F10-IgG1-hole plasmid in a 1:1 ratio, with PEImax (1 µg/µl) in a 3:1 ratio in OptiMEM. Supernatant was harvested at day 6, centrifuged (30 minutes, 4000 rpm) and filtered using 0.22 µm Steritop filters (Merck Millipore). Antibodies were purified using protein A/G (Pierce) affinity chromatography. Antibodies were eluted from the column using 0.1 M glycine (pH 2.5) and neutralized using neutralization buffer 1M Tris (pH 8.7). The eluates were concentrated and buffer exchanged to PBS using 50 kDa Vivaspin filters (GE Healthcare). In all assays we correct for protein size using the following calculation: $C=\frac{m}{V}x\frac{1}{MW}$ . Where C is the molar concentration in mol/L or M, m is mass of the protein in grams (g), V is volume of solution in liters (L) and MW is the molecular weight of the protein in g/mol.

### Protein Design and Purification

All HIV-1 Env SOSIP.v9.0 trimers (CNE55, Ce1176, 25710, CH119.10, BJOX002000.03.2, Ce7030 and 246-F3_C10) contain a disulfide bond to covalently link gp120 to the gp41 ectodomain and an amino acid substitution to strengthen interactions between the gp41 subunits, creating a good resemblance of native virus spikes (33). BG505 gp120 and other construct were cloned into a pPPI4 expression vector and produced as described before (34). In short, SOSIP.v9.0 trimers (35) were transiently expressed in the presence of furin expression plasmid (ratio 4:1) into HEK-293F cells (Invitrogen). Supernatant was harvested at day 6, centrifuged (30 minutes, 4000 rpm) and filtered using 0.22 µm Steritop filters (Merck Millipore). For purification, columns made from PGT145 or PGT151 bNabs coupled to CNBr-activated Sepharose 4B beads were used, as described earlier (34). The supernatants were passed over the column by gravity and subsequently washed twice with buffer (0.5 M NaCl, 20 mM Tris, pH 8.0). The SOSIP.v9.0 trimers were then eluted with 3M Mg_2_Cl_2_ pH 7.8, directly into neutralization buffer (20 mM TrisHCl pH8.0, 75 mM NaCl). After purification Env proteins were concentrated using Vivaspin 10 or 100 kDa filters (GE healthcare). For JRCSF gp41, a gene corresponding to amino acids 543–665 in HXB2 numbering was cloned into a pPPI4 plasmid containing a hexahistidine tag. Transfections were performed using the expression plasmids and 1 µg/µl PEImax (Polysciences) in a 3:1 ratio in OptiMEM. The His-tagged protein was purified from the supernatant with affinity chromatography using NiNTA agarose beads (QIAGEN). Further purification was performed using size-exclusion chromatography on a Superdex HiLoad 16/600 column (GE Healthcare) using PBS as buffer. Avi-tagged proteins were biotinylated using the BirA500 biotin-ligase reaction kit (Avidity) according to the manufacturer’s protocol.

### SDS-PAGE

The nanobodies and nanobody-IgG1 constructs were analyzed using SDS-PAGE followed by Coomassie blue dye staining. 5 µg protein was mixed with loading dye (0.125 M Tris-HCl pH 6.8, 20% glycerol, 4% SDS, 0.05% bromephenol blue in milli-Q water) and dithiothreitol (DTT; 50 mM) and incubated at 95°C for 10 minutes prior to loading on a 4–12% Tris-Glyine gel (Invitrogen). Precision Plus Protein Standard Dual Color (Biorad cat#161-0374) was used as a marker. The gel was run for 2 hours at 125V in running buffer (25 mM Tris, 192 mM glycine, 0.05% SDS). After running, gel was stained using the PageBlue Protein Staining Solution (Thermo Scientific) for 60 minutes. Gel was destained using ultrapure water with gentle agitation.

### Neutralization Assay

Neutralization assays were performed as described previously (34). In brief, TZM-bl cells were seeded (DMEM, 10% FCS, 1/1000 P/S) to achieve a confluency of 70–80%. Virus mix was added to the antibodies to allow binding for 1 hour at RT. Dextran (DEAE) and Saquinavir (SQV) were added to the cells in a final concentration of 40 μg/ml and 400 nM respectively. Afterwards, the virus+antibody mix was added to the cells and incubated at 37°C for 72 hours. After incubation, the cells were lysed using 1x lysis buffer and incubated on an orbital shaker for 20 minutes at RT. Afterwards, 5 µl lysate and 25 µl Bright Glo Luciferase was added to a white 96 well plate and subsequently luciferase activity of cell lysate was measured using a Glomax plate reader. The inhibitory concentration (IC_50_) was determined as the concentration of mAb were 50% of the virus was neutralized using a dose response non-linear regression fit in GraphPad Prism 9.

### Bio-Layer Interferometry

Bio-layer interferometry was used to characterize antibody-antigen interactions. Biotinylated BG505 SOSIP.664 or JRCSF gp41 was loaded on streptavidin sensors (Sartorius), washed using running buffer [PBS, 0.02% Tween 20, and 0.1% bovine serum albumin (BSA)] to remove excess protein. This was followed by the transfer to a well containing nanobodies or nanobody-IgG1s (10 nM-1.12 nM) in running buffer to measure association. The biosensors were then dipped in running buffer to measure dissociation of the antibody-antigen complexes. For the characterization of the bispecific antibodies, the biosensor was subsequently dipped in a well containing a second protein (BG505 gp120 or 93IN905 gp120 in running buffer), followed by a well containing running buffer to measure dissociation. The biosensors were dipped in regeneration buffer (PBS, 10 mM glycine) and into activation buffer (PBS, 10 mM NiCl_2_) before measuring new antibody-antigen complexes. Assays were performed at 30°C and association and dissociation were measured for 300 seconds. All measurements were performed using an Octet K2 (ForteBio).

### Surface Plasmon Resonance FcRn

Biotinylated FcRn was spotted sextuple in 3-fold dilutions, as previously described (36), ranging from 30 nM to 1 nM onto a single SensEye G-streptavidin sensor (Ssens, 1–08–04–008) using a Continuous Flow Microspotter (Wasatch Microfluidics). Spotting was done in PBS supplemented with 0.075% Tween-80 (VWR, M126–100ml), pH 7.4. The spotted sensor was then placed in an IBIS MX96 (IBIS technologies) device to measure the SPR signals. The different antibodies were then injected at a 2 times dilution series starting at 0.12 nM until 125 nM in PBS + 0.075% Tween-80, pH 6. Regeneration was carried out after every sample with a two times pulse of 12 seconds with 20 mM Tris + 150 mM NaCl, pH 8.8. Calculation of the dissociation constant (K_D_) was performed by equilibrium fitting to R_max_ = 1000 RU using a power fit using the K_D_ and corresponding R_max_ of all the spotted biotinylated FcRn. Analysis and calculation of all binding data was carried out with Scrubber software version 2 (Biologic Software) and Excel.

### Binding ELISA

ELISAs were performed as described previously (32). ELISA plates were coated, overnight at 4°C, with *Galanthus nivalis* lectin (Vector Laboratories) in 0.1 M NaHCO_3_. The next day, the plates were washed 3 times with 1X Tris-buffered saline (TBS). The washing step was repeated after each incubation step. Residual binding was blocked using 50 µl 1% (w/v) Casein in TBS (Thermofisher) for 30 minutes at RT before coating plates with recombinant SOSIP.v9.0 trimers (2 µg/ml) in casein in TBS (2 hours, RT). Next, plates were incubated with serial antibody dilutions for 2 hours at RT. After antibody incubation, the plates were incubated with horseradish peroxidase labeled secondary antibody (goat anti-human IgG 1:3000 or anti-VHH-HRP (Genscript) 1:5000) in 1% Casein in TBS for 1 hour at RT. Subsequently, plates were washed 5 times with 1X TBS/0.05% Tween-20. Develop solution (0,1 M NaAc + 0,1 M citric acid + 1% TMB + 0,01% H_2_O_2_) was added and 0.8 M H_2_SO_4_ was used to stop the reaction after 2 minutes. Optical density was measured using a SPECTROstar Nano Microplate Reader (BMG LabTech) with a 450nm filter.

### FcγR Dimer ELISA

ELISA plates were coated overnight with *Galanthus nivalis* lectin (Vector Laboratories) in 0.1 M NaHCO_3_. The next day, the plates were washed 3 times with 1X TBS. Plates were coated with Ce1176 SOSIP.v9.0 trimer (2 µg/ml) in 1X DPBS for 2 hours at 37°C. After incubation, the plates were washed 4x using TBS-0.05% Tween-20 (TBS-T) and blocked by Assay buffer (1% BSA, 0.05% Tween-20, 1mM EDTA in PBS) for 1 hour at 37°C. After blocking, the plates were washed 4x using TBS-T. Next, plates were incubated with serial antibody dilutions for 2 hours at 37°C. After incubation, plates were washed and incubated with biotinylated FcγRIIIa or FcγRIIa dimers (0,5 µg/ml) in assay buffer for 1 hour at 37°C. FcγRIIIa and FcγRIIa dimers, described previously (37), were produced in HEK-293F cells using PEImax, purified using NiNTA columns (Qiagen) and biotinylated using the Biotin-protein ligase kit (Sigma-Aldrich). The correct protein fraction was separated by size-exclusion chromatography using a superose200 column (Cytiva). Subsequently, the plates were washed and incubated with high sensitivity streptavidin-HRP detection antibody 1:2,000 (Biolegend) in Assay Buffer for 1 hour at 37°C. Following incubation, plates were washed 4x with TBS-T. Develop solution (0,1 M NaAc + 0,1 M citric acid + 1% TMB + 0,01% H_2_O_2_) was added and 0.8 M H_2_SO_4_ was used to stop the reaction. Optical density was measured using a SPECTROstar Nano Microplate Reader (BMG LabTech) with a 450nm filter.

### Binding to Env Expressing Cells

HEK-293F cells (Invitrogen) were transfected using BG505 gp160 pCI plasmid expression vector and lipofectamine (thermofisher) in OptiMEM as previously described (38). After 48 hours, HEK-293F were harvested and opsonized for 1 hour at 37°C with serial dilutions of the nanobody-IgG1 antibodies. After incubation, the cells were washed twice using PBS 2%FCS and subsequently stained for 30 minutes at 4°C with Goat F(ab’)2 anti-Human IgG-PE (Southernbiotech) and analyzed using flow cytometry.

### Antibody-Dependent Cellular Trogocytosis (ADCT)

BG505-gp160 expressing HEK-293T cells, previously described, were stained with 10 µM PKH26 dye (Sigma-Aldrich) and incubated for 20 minutes with periodic mixing. THP-1 cells (ATCC) were stained intracellularly with 0.5 µM CFSE (Thermo Fisher Scientific) in PBS and incubated for 20 minutes with periodic mixing. Both target and effector cells were washed twice with PBS and taken up in culture medium. The stained HEK-293T cells were opsonized for 30 minutes at 37°C with serial dilutions of the nanobody-IgG1 antibodies. After incubation, cells were washed and THP-1 cells were added to the HEK293T cells at a 2:1 effector:target ratio. Plates were quickly spun down to promote cell to cell contact before incubation for 1 hour at 37°C. Afterwards, cells were washed and resuspensed in PBS 2% FCS. Flow cytometry was used to measure the double positive, PKH26+ CFSE+, THP-1 cells. ADCT was calculated by the fluorescent PKH26 signal of the THP-1 cells. To determine the background, trogocytosis in the absence of antibody was measured.

### Antibody-Dependent Cellular Phagocytosis (ADCP)

Fluorescent Neutravidin beads (Invitrogen) were incubated with biotinylated CNE55 SOSIP.v9.0 trimer (5µg/10 µl beads) overnight at 4°C. Beads were subsequently spun down and washed twice in PBS containing 2% bovine serum albumin (BSA) to remove unbound antigen and block the remaining hydrophobic sites on the microspheres. The coated beads were resuspended in PBS 2% BSA at a 1:500 dilution. 50 μl of the beads suspension was placed in every well of a V-bottom 96-well plate and incubated for 2 hours at 37°C with serial dilutions of the nanobody-IgG1 antibodies. After incubation, plates were washed and 5×10^4^ THP-1 effector cells (ATCC) were added to each well in a final volume of 100 μl. Subsequently, plates were quickly spun down to promote beads to cell contact before incubation for 5 hours at 37°C. After incubation, the cells were washed, resuspended in PBS 2% FCS and analyzed by flow cytometry. Phagocytic activity was determined by the area under curve of the MFI (beads positive cells x mean MFI FITC). The data is represented as area under the curve for threefold titrations from 1–0,01 µg/ml for all antibodies.

### NK-Activation Assay

Peripheral blood monoculear cells (PMBCs) were isolated from buffy coats (Sanquin) by using Ficoll-Paque (Cytiva) according to the manufacturer’s instructions. Subsequently the natural killer (NK) cells were isolated using positive selection MicroBeads against CD56 (Macs Miltenyi, Biotec). In short: cells are labeled with CD56 MicroBeads and loaded onto a MACS Column which is placed in a magnetic field. The cells positive for CD56 are retained within the column and are eluted using PBS, pH 7.2, 0.5% (m/v) BSA, and 2 mM EDTA. BG505 gp160 transfected HEK-293T cells were used as target cells. NK cells were stimulated with IL-15 (10 ng/ml, o/n, 37°C). The next day, the plates were washed 3 times with 1X TBS. Plates were coated with Ce1176 SOSIP.v9.0 trimer (2 µg/ml) in PBS for 2 hours at 37°C. After incubation, plates were blocked with PBS-1% BSA for 1 hour at 37°C and thereafter washed 3x with TBS. Plates were incubated with serial dilution series of antibodies in PBS-1% BSA for 2 hours at 37°C. After incubation, the plates were washed 3x with TBS and then incubated with 50 µl IL-15 stimulated NK cells (50.000 cells/well) for 3 hours at 37°C. Then, the NK cells were transferred to a 96-well V-bottom plate, washed using FACS buffer (2% FCS, PBS) and stained for anti-CD16-PE (Biolegend) and anti-CD107a-APC (Biolegend). Cells were analyzed using flow cytometry and percentage CD107 and CD16 positive NK-cells were determined.

## Results

### Multivalent Anti-HIV-1 Nanobodies Show Increased Binding Towards Their Target Epitope

**Figure 1:**
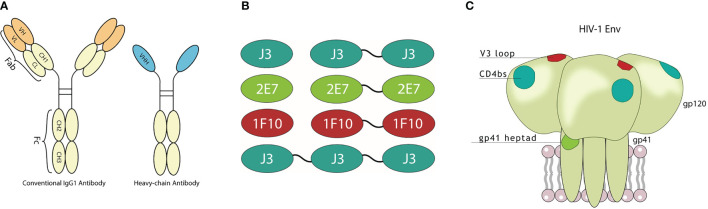
Visualization of nanobodies and their target epitope. **(A)** Schematic representation of a conventional immunoglobulin (IgG) antibody and a camelid heavy-chain only antibody with visualization of their variable domains. **(B)** Monovalent, bivalent and trivalent J3 targeting the CD4bs, monovalent and bivalent 1F10 targeting the V3 loop and monovalent and bivalent 2E7 targeting the gp41 heptad repeat-1 (HR1). **(C)** Schematic diagram of the HIV-1 trimeric envelope glycoprotein complex showing the epitopes that are recognized by the broadly neutralizing nanobodies.

**Figure 2:**
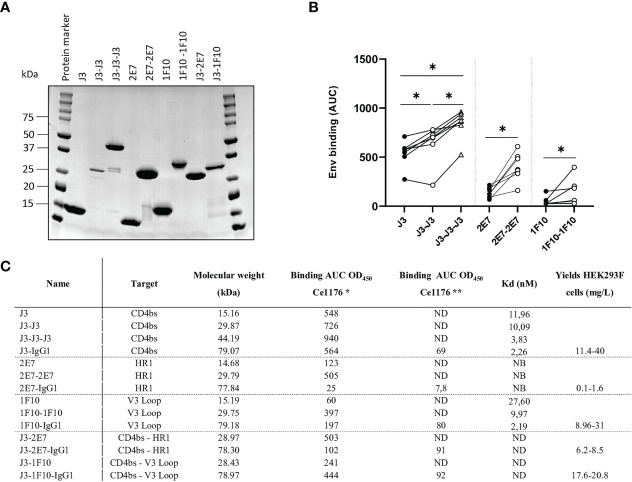
Multivalent anti-HIV-1 nanobodies show increased binding towards HIV-1 Env trimers from global panel. **(A)** SDS-PAGE protein separation followed by coomassie blue staining of the purified anti-HIV-1 nanobody-constructs under reducing conditions. Molecular weight (MW) is indicated in kilodalton (kDa). **(B)** Average area under the curve (AUC) of binding curves of J3, 2E7 and 1F10 nanobody variants to SOSIP.v9.0 trimers from the global panel (CNE55, Ce1176_A3, 25710-2.43, CH119.10, BJOX002000.03.2, Ce7030 and 246-F3_C10) as determined by ELISA using OD_450_. Average AUC of OD450 was determined by doing a definite integral between the start and end point using GraphPad. Friedman matched comparison (three groups) and Wilcoxon matched-pairs signed rank tests (two groups) was used to compare the groups with significance indicated as *p <0.05. Data shown are the average of three independent experiments. **(C)** Binding of nanobodies and nanobody-IgG1 constructs to Ce1176 as determined by ELISA. *The binding of the nanobody and nanobody-IgG1 constructs to the concerned epitopes was determined using an anti-VHH-HRP secondary antibody, starting concentration 500 nM.** The binding of the nanobody-IgG1 constructs to the concerned epitopes was determined using a Goat-anti-Human-HRP secondary antibody, starting concentration 50 nM. Kd values for BG505 SOSIP.664 as determined by BLI, based on the average association of different antibody concentrations. ND, not determined; NB, non-binding. Data are representative of at least two independent experiments.

We constructed bivalent and trivalent versions of anti-HIV-1 Env nanobodies J3, 2E7 and 1F10, targeting the CD4bs, HR1 and V3 loop respectively, using flexible glycine-serine (GS) linkers (**Figure 1**) to enhance binding to the HIV-1 Env trimer. Purity and correct size of the multivalent nanobodies was confirmed with a reduced SDS-PAGE gel (**Figure 2A**). Next, we assessed binding of the multivalent nanobodies to recombinant SOSIP.v9.0 trimers from the global panel. The bivalent nanobodies displayed enhanced binding to the tested recombinant SOSIP.v9.0 trimers compared to their monovalent counterpart (**Figure 2B**). In addition, trivalent J3 showed increased binding compared to monomeric and bivalent J3. Interestingly, while a monovalent 1F10 showed no binding to most SOSIP.v9.0 trimers, a bivalent 1F10 gained the ability to bind these targets (**Figure 2B**, **S1**). This was supported by biolayer interferometry (BLI) results which showed that J3 and 1F10 multivalent nanobodies had a higher affinity for BG505 SOSIP.664 (**Figure 2C**, **S2**). Thus, multivalent nanobody constructs bind stronger to HIV-1 Env compared to their monovalent counterparts.

### Bivalent and Trivalent Nanobodies Show Enhanced Neutralization Potency

**Figure 3:**
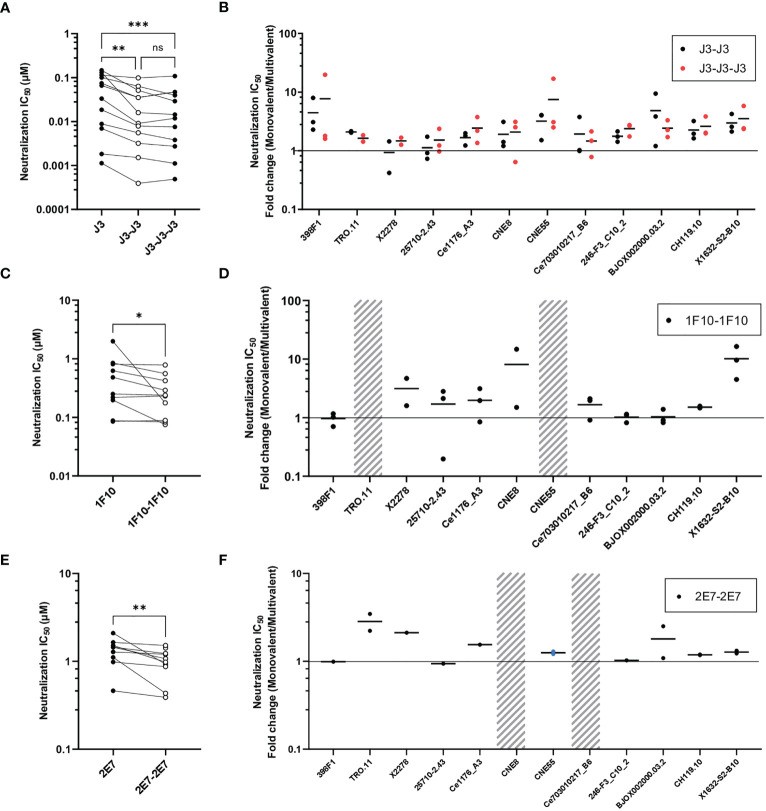
Multivalent anti-HIV-1 nanobodies show enhanced neutralization capacity depending on target. **(A)** Neutralization IC_50_ (µM) of J3 variants against 12 viruses from the global panel. **(B)** Neutralization capacity of bivalent (black dots indicate replicates) and trivalent J3 (red dots indicate replicates) as compared to monovalent J3 is depicted as fold change in IC_50._ **(C)** Neutralization IC_50_ (µM) of 1F10 variants against 12 viruses from the global panel. **(D)** Neutralization capacity of bivalent 1F10 as compared to monovalent 1F10 is depicted as fold change in IC_50_. **(E)** Neutralization IC_50_ (µM) of 2E7 variants against 12 viruses from the global panel. **(F)** Neutralization capacity of bivalent 2E7 as compared to monovalent 2E7 is depicted as fold change in IC_50._ Blue dots indicate neutralization by bivalent nanobody but not by monovalent nanobody. Absence of neutralization (IC_50_ > 1 or 1.5 µM) is indicated with a grey shade. Friedman matched comparison (three groups) and Wilcoxon matched-pairs signed rank tests (two group) was used to compare IC_50_. Significance is indicated as *p < 0.05, **p < 0.005, ***p < 0.0005 and “ns” indicates not significant (p > 0.05). Data shown are the average of two or three independent experiments.

Next, we studied the neutralization capacity of the multivalent nanobodies against viruses from the global panel, to determine whether the increase in binding also translates to more potent neutralization (**Figure 3**). In line with previous studies, J3 neutralized all strains potently with an average IC_50_ of 0.06 µM. Trivalent J3 showed significantly improved neutralization potency (> 2 fold change) compared to the monovalent counterpart for all viruses from the global panel. Bivalent J3, however, showed significantly improved neutralization to all isolates compared to monovalent J3, except against X2278 and 25710-2.43, where the average fold change was below 2 (**Figure 3A**, **S3**). In general, the neutralization capacity of trivalent J3 across the global panel was similar to that of bivalent J3 (**Figure 3B**), suggesting that the advantage of a third nanobody domain in terms of binding is not translated to enhanced neutralization capacity. Bivalent 1F10 showed significantly improved neutralization against viruses from the global panel compared to the monovalent variant (**Figure 3C**), with the greatest improved neutralization against the virus X1632-S2-B10 (**Figure 3D**). Though previous ELISA results showed that 1F10 nanobodies did not bind Ce7030 and BJOX002000.03.2, they showed neutralization of these viral strains, indicating that the V3 loop is more accessible on pseudoviruses compared to SOSIP.v9.0 trimers. Bivalent 2E7 showed significantly increased neutralization potency against viruses from the global panel compared to the monovalent variant (**Figure 3E**). Generally, bivalent 2E7 induced a similar enhancing trend across the whole global panel (**Figure 3F**). Interestingly, while monovalent 2E7 was unable to neutralize CNE55, bivalent 2E7 showed neutralization with an IC_50_ of 1.4 µM. Overall, the increase in neutralization potency seems to correlate with the increase in binding observed previously. This suggests that the increased avidity by linking nanobodies together to create multivalent structures not only increases antigen-binding but also enhances neutralization potency.

### Design and Production of Nanobody-IgG1 Constructs Targeting HIV-1

**Figure 4:**
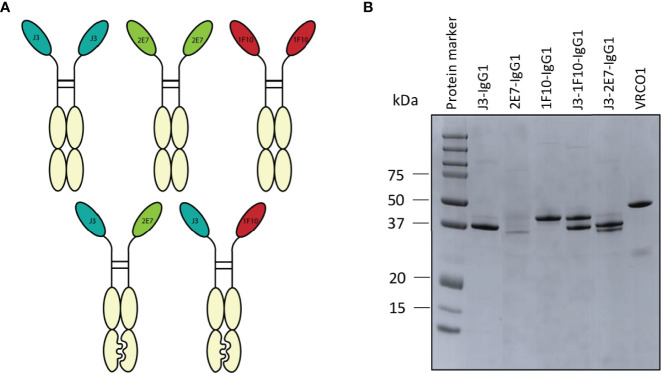
Creation of nanobody-IgG1 constructs and their ability to bind to their target epitope. **(A)** Representation of the different nanobody-IgG1 constructs. 2E7, 1F10 and J3 fused to an human IgG1 heavy chain, lacking the CH1 domain and the bispecific J3-1F10 and J3-2E7 antibodies containing the S354C, T366W, K409A, Y349C, T366S, L368A, Y407V, F405K knob in hole and electrostatic mutations. **(B)** Visualization of the anti-HIV-1 nanobody-IgG1 fusions and the conventional IgG1 VRC01 under reducing conditions. Molecular weight (MW) is indicated in kDa.

The main goal of the IgG1 Fc fusion was to gain Fc-dependent effector functions that contribute to the killing of HIV-1 infected cells. Furthermore it would create bivalent molecules with increased half-life. Two of each nanobodies J3, 2E7 or 1F10 were directly fused to the hinge of an IgG1 Fc domain to create nanobody-IgG1 proteins (**Figure 4A**). These nanobody-IgG1 constructs were successfully produced in HEK-293F cells and purified after which molecular weight and the composition of the constructs was confirmed using PAGE (**Figure 4B**). Most nanobody-IgG1 constructs resulted in relatively pure proteins with acceptable yields, except for 2E7-IgG1. The production of 2E7-IgG1 being only successful in larger volumes, indicates that the construction or folding of 2E7-IgG1 might not be optimal by HEK-293F cells. Validation of bispecific antibodies was done using SDS-PAGE, which showed that the bispecific nanobodies were produced with the correct molecular weights, corresponding to two nanobody-IgG1s (**Figure 4B**). Furthermore, BLI was used to confirm bispecificity of the constructs and to confirm that all the different nanobody domains in these constructs could still bind to their cognate HIV-1 Env epitopes. We utilized three different proteins that were only recognizable by either J3, 1F10 or 2E7 (**Supplementary Figure 4**). This confirmed that our bispecific nanobody-IgG1s are indeed bispecific and not a mixture of the corresponding nanobody-IgG1s and that ability to bind their target epitope was retained. As assessed by ELISA, the generated nanobody-IgG1s remained functional and retained the ability to bind the Ce1176 SOSIP.v9.0 trimer (**Figure 2C**). Only 2E7-IgG1 showed decreased binding compared to its nanobody variants, likely caused by the lower antibody quality.

### Neutralization Potency of Nanobody-IgG1 Constructs

**Figure 5:**
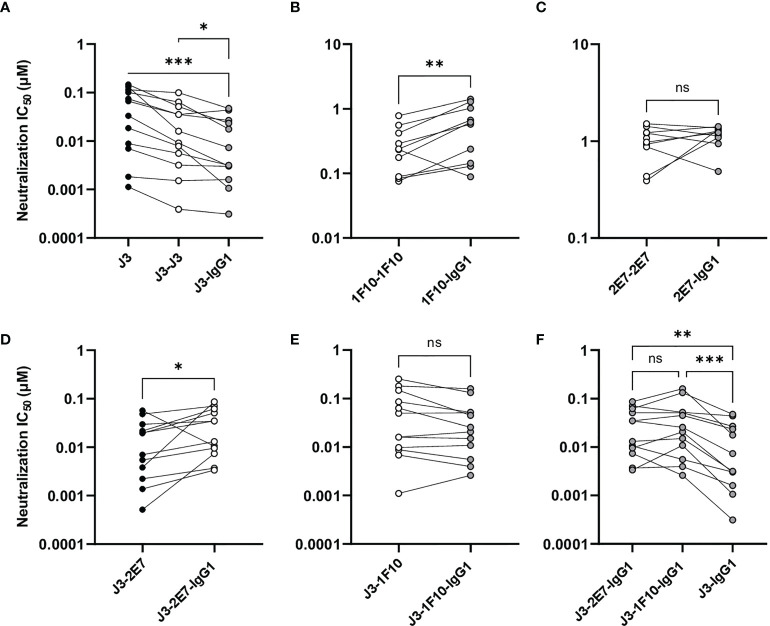
Nanobody-IgG1 constructs are able to induce Fc-mediated effector functions. Neutralization capacity of IgG1 fusion constructs compared to monovalent or bivalent versions of **(A)** J3 **(B)** 1F10 and **(C)** 2E7 and **(D)** J3-2E7 and **(E)** J3-1F10. **(F)** Neutralization capacity of J3-IgG1 compared to bispecific J3-2E7-IgG1 and J3-1F10-IgG1. Friedman matched comparison was used to compare neutralization IC_50_ (µM) between three groups. Wilcoxon matched-pairs signed rank tests was used to compare neutralization IC_50_ (µM) between two groups. Significance is indicated as *p <0.05, **p <0.005, ***p <0.0005 and not significant (ns). Data shown are the average of three independent experiments.

Next, we studied the neutralization capacity of the nanobody-IgG1s against viruses from the global panel. J3-IgG1 showed increased neutralization potency to most viruses of the global panel compared to the monovalent and bivalent nanobody (**Figure 5A**). 1F10-IgG1, however, showed a decrease in neutralization capacity against all tested viruses when compared to the bivalent nanobody variant, except to the virus CNE8 (**Figure 5B**, **S5**). The fusion of 2E7 to an IgG1 Fc domain did not result in significant changes in neutralization capacity against viruses from the global panel (**Figure 5C**). Taken together, these experiments showed that the nanobody-IgG1 fusion was successful, although it can impact the binding to the target epitope. Moreover, these results suggest that the orientation and flexibility of the nanobody domains determines Env recognition and neutralization capacity, and that the influence of the Fc domain on neutralization is epitope and virus dependent.

### Bispecific Nanobody-IgG1 Constructs Did Not Result in Enhanced Neutralization Potency

A previous study showed that neutralization of HIV-1 strains can be enhanced by the creation of bispecific nanobodies (23, 39, 40). J3 showed to be the broadest and most potent nanobody and 2E7 and 1F10 could both neutralize two viral strains that J3 did not. Therefore we constructed bispecific nanobodies, consisting of J3-2E7 and J3-1F10, as well as bispecific IgG1 variants using knob-in-hole and electrostatic mutations (41) (**Figure 4A**). Next, we studied the neutralization capacity of these bispecific nanobodies and IgG1 constructs against viruses from the global panel. In line with previous studies, the bispecific nanobodies neutralized viruses from the global panel quite potently (**Supplementary Figure 3**). We further found that the bispecific nanobodies enhanced neutralization compared to a mixture of the corresponding nanobodies, while for the nanbody-IgG1s no improvement was found (**Supplementary Figure 6**). Furthermore, the fusion of J3-2E7 to an Fc domain led to a decrease in neutralization potency, whilst J3-1F10-IgG1 resulted in no change in neutralization potency compared to the bispecific nanobody variant (**Figures 5D, E**). In addition, the neutralization by J3-IgG1 was more potent than both J3-1F10-IgG1 or J3-2E7-IgG1 against the tested viruses from the global panel, suggesting that the second J3 head contributes substantially to potent neutralization (**Figure 5F**). Overall, these results show that that the knob-in-hole mutations do not impact binding and that changes observed in neutralization are likely attributed to the IgG1 format, the distance and flexibility between the nanobody domains in the bispecific constructs.

### Nanobody-IgG1 Molecules Induce Antibody-Dependent Effector Functions

**Figure 6:**
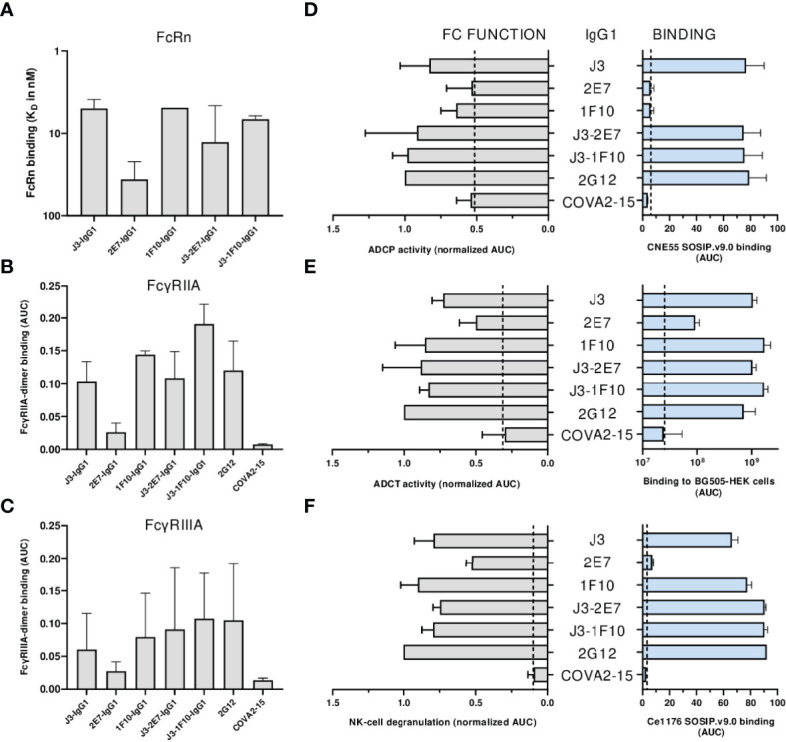
Neutralization is impacted by nanobody-IgG1 fusion. **(A)** The K_D_ of all nanobody-IgG1s to hFcRn, recorded at pH 6.0 using surface plasmon resonance. K_D_ was calculated as the average K_D_ from multiple antibody concentrations as seen in **Supplementary Figure 5**. Binding of nanobody-IgG1s to **(B)** FcγRIIa and **(C)** FcγRIIIa as determined by FcgγR dimer ELISA using Ce1176 SOSIP.v9.0 as coating antigen, depicted as AUC values of the binding curves. **(D)** Binding of nanobody-IgG1s to CNE55 SOSIP.v9.0 as determined by ELISA and induction of ADCP using CNE55 SOSIP.v9.0 coated fluorescent beads, both depicted as AUC. **(E)** Binding of nanobody-IgG1s to BG505 gp160 expressing HEK-293T cells and induction of ADCT are quantified by a secondary PE-labeled anti-IgG antibody and uptake of membrane fragments by THP-1 cells using flow cytometry, both depicted as AUC. **(F)** The binding strength for all nanobody-IgG1s to Ce1176 SOSIP.v9.0 as determined by ELISA and subsequent activation of NK-cells plotted as the percentage of CD107+ NK-cells. 2G12-IgG1, specific for HIV-1 gp120, was used as a positive control and COVA2-15, specific for SARS-CoV-2, was used as a negative control. The dotted lines represent the background level that is observed in these assays in the absence of antibody. Effector function data is normalized to the positive control, 2G12. Data shown are the average of at least two independent experiments.

First the binding of the nanobody-IgG1s to the neonatal Fc receptor (FcRn) and Fc-gamma receptors (FcγRs) was assessed. All nanobody-IgG1s were able to bind to FcRn and FcγRIIA, FcγRIIIa with similar affinity as compared to a conventional IgG1 bNab (**Figures 6A–C**, **S7**). Except for 2E7-IgG1, which was found to bind less strong to all FcRs. Next, the induction of ADCP by nanobody-IgG1 antibodies was measured using CNE55 SOSIP.v9.0 coated beads and THP-1 cells. We found that when opsonized with the nanobody-IgG1s, the beads were phagocytosed by the activated THP-1 cells in a dose-dependent manner (**Figure 6D**). Furthermore, all the nanobody-IgG1s had the ability to induce ADCT shown by the increase of PKH26 on THP-1 cells when incubated with opsonized BG505 gp160 transfected HEK-239T cells (**Figure 6E**). Lastly, it was evaluated whether the nanobody-IgG1s could induce activation of NK cells after binding to Ce1176 SOSIP.v9.0, a clade C trimer, using CD107a as a marker of degranulation (**Figure 6F**). All nanobody-IgG1s were able to activate NK cells, indicating the potential of these construct to induce ADCC-mediated cell death. In general, binding by the nanobody-IgG1s to their target protein correlated with effector function induction, hence why 2E7-IgG1 showed lower induction of these effector functions as the binding was less (**Supplementary Figure 8**). These results demonstrate that the fusion of nanobodies to an human IgG1 Fc domain can result in the ability to mediate effector functions with similar potencies as a conventional IgG1 bNab.

## Discussion

Anti-HIV-1 nanobodies display advantages over conventional bNAbs, including stability, homogeneity, small-size and capacity to bind cryptic epitopes on the HIV-1 Env trimer. Moreover, they have been shown to efficiently neutralize HIV-1 with high potency and breadth. Therefore these nanobodies are interesting candidates as alternative HIV-1 treatment options or possibly even for new therapeutic approaches to eradicate HIV-1 from people living with HIV-1, for example with the shock-and-kill strategy. We extended the functionality and applicability of three anti-HIV-1 nanobodies (J3, 2E7 and 1F10) as therapeutic agent: by developing multivalent constructs and nanobody-IgG1 constructs.

In general, the design of multivalent anti-HIV-1 nanobodies improved binding to the HIV-1 Env trimer and enhanced neutralization potency (42, 43). The enhanced avidity is likely to be explained by inter-spike crosslinking and not by intra-spike crosslinking, as the distance to another protomer on the same trimer is far greater than the distance to an adjacent trimer (44). It is expected that the flexible linker in the bivalent constructs is too short for intra-spike crosslinking due to the architectural structure of the Env, while for the bispecific constructs this length could be sufficient as both epitopes reside closer together. Naturally, the linker length connecting the anti-HIV-1 nanobodies has to allow for both heads to bind the HIV-1 Env trimer simultaneously, either on the same trimer or on two adjacent trimers. Our GS linker length was previously optimized for each multivalent anti-HIV-1 nanobody on the basis of Env binding in ELISA. It would be insightful to use more advanced techniques such as Cryo-EM to study the optimal linker length for each multivalent nanobody for intra-spike binding. For example, the optimal linker length for intra-spike crosslinking for a bispecific scFv targeting the CD4bs and V3-glycan was determined using Cryo-EM (39). Further optimization of linker length will enhance the potential implementation of multivalent nanobodies as therapeutic agent.

The nanobody-IgG1 constructs are considerably larger than the original nanobody which influenced the binding and neutralization properties. Fusion of the IgG1 Fc domain to 2E7 was found to decrease binding capacity and neutralization potency, indicating that the larger size was interfering with Env binding. The mode of action of 2E7 is similar to monoclonal Abs (mAbs) binding an epitope located at the N-terminal part of the HR1. The breadth and potency of these mAbs is largely increased when used as smaller single chain antibodies or Fabs, indicating that the HR1 epitope is difficult to access for conventional antibodies during the fusion reaction (45–47). This could explain why neutralization was impacted less than binding, as the epitope might become more accessible for 2E7-IgG1 after binding to CD4 (48). While the Fc domain could negatively impact binding, it could also aid with the prevention of membrane fusion. Recently a nanobody targeting the chemokine receptor CXCR4 was fused to an IgG Fc domain, which resulted in inhibited HIV-entry (49). Similar results were found for J3-IgG1, which showed enhanced neutralization potency compared to bivalent J3, likely caused by the bivalent structure or by increased steric hindrance.

Improvement of neutralization capacity by the Fc fusion constructs is likely dependent on nanobody flexibility as well as epitope location and accessibility. The IgG1 hinge domain restricts flexibility while binding of both heads to the Env spike is only possible when the epitopes are in close proximity (50). Though J3-IgG1 showed enhanced potency, 1F10-IgG1 led to decreased potency, indicating that the orientation and the distance between the nanobody heads is more optimal for reaching two CD4bs than for reaching two adjacent V3 loops. Additionally, results for 1F10 seemed viral strain dependent. This can be caused by antigenic variations of the V3 region and specific residues outside of V3, which can affect the presentation of the epitope hereby restricting access for antibodies (51–53). While V3 loop exposure has not been extensively studied, it is known that the V3 loop is shielded by several N-glycans and remains largely buried, and its accessibility is improved after conformational changes (54–56). Therefore, in contrast to the CD4bs, it is rather unlikely that all three V3 loops on the Env trimer are exposed for efficient bNab targeting.

The nanobodies were fused to an IgG1 Fc domain to create bivalency, increase half-life *via* FcRn interaction and at the same time install Fc dependent functions which are essential for the killing of HIV-1 infected cells as part of the shock-and-kill strategy. These Fc dependent functions contribute to a delay in HIV acquisition, disease progression and help suppress viremia and aid with the clearance of infected cells (14, 57, 58). All constructs contained the same Fc region, indicating that differences in signaling through FcγRs to facilitate effector functions is dependent on their variable region, binding affinities and accessibility of the Fc region when bound to the target. This corresponds with a study where a significant correlation between the binding of an antibody to the cell and their ability to induce ADCC was found (59). Nanobodies have improved neutralization capacities due to accessibility of the precluded neutralizing epitopes with their small size and long CDR3 (21, 60). However, the lack of Fc region prevents them from mediating killing of HIV-1 infected cells. On the contrary, nanobody-IgG1s have the ability to broadly neutralize viral strains and to mediate target cell killing, making these constructs interesting candidates for the kill in a shock-and-kill strategy. Further studies should focus on whether these constructs are able to kill latently infected immune cells in combination with latency reversal agents. In addition, Fc-engineering approaches that proved successful for conventional antibodies might also improve the engagement and activation of effector cells by nanobody-IgGs (58, 61, 62).

To increase breadth and reduce the emergence of viral escape mutants, targeting two epitopes on Env is likely to be beneficial. Therefore, bispecific constructs consisting of J3-1F10 and J3-2E7 were created. In contrast to previous data (23), both constructs did not enhance neutralization potency compared to their nanobody variant or J3-IgG1. This indicates that either binding of each nanobody head affects binding of the other, that the Fc domain restricts flexibility or that a second J3 head attributes significantly more to potent neutralization. A recent study coupled a gp120 and a gp41 targeting protein together using a 35-mer linkers, which resulted in good anti-HIV-1 activity (63). We could opt to fuse the bispecific nanobodies, with more possibilities to adjust linker length for optimal epitope reach directly to the hinge of the IgG1, creating a tetravalent construct. Although bispecific J3-2E7-IgG1 and J3-1F10-IgG1 did not show improvement in neutralization potency compared to J3-IgG1, the IC_50_ values are similar and still in nM ranges. Because of their bispecificity and functional Fc domain, they would make a great tool that can be further improved for anti-HIV therapies with high neutralization breadth.

In summary, we have generated potent multivalent anti-HIV-1 nanobodies and functional nanobody-IgG1s. We show that binding and neutralization potency can be enhanced by creating multivalent nanobodies by means of increased binding to the Env trimer. Moreover, fusion to an IgG1 domain enhanced neutralization capacity, whilst being dependent on epitope location and accessibility, and allowed for Fc-mediated activation of immune cells. These engineered anti-HIV-1 nanobody constructs have shown their therapeutic potential and may prove to be valuable towards HIV-1 prevention, treatment, or cure strategies.

## Data Availability Statement

The raw data supporting the conclusions of this article will be made available by the authors, without undue reservation.

## Author Contributions

AS, MJG and ST contributed to conception and design of the study. AS performed most experiments and analyzed the data. AB carried out FcRn binding using SPR. RH and GD provided the purified nanobodies. MH and MP were responsible for production of the SOSIP proteins. TG, NK, RS, TV, and GV helped with funding and resources. AS wrote the first draft of the manuscript. ST and MJG helped review manuscript. All authors contributed to manuscript read and approved the submitted version.

## Funding

This work was supported by HealthHolland/Aidsfonds: LSHM19101/P-44802, by HealthHolland/AMC: 2019-1167 and the AMC Fellowship from Amsterdam UMC received by MJG. ST is supported by Young investigator grant P-53301 Aidsfonds.

## Conflict of Interest

Authors GD and RH were employed by QVQ Holding BV. Author TV was employed by VerLin BV.

The remaining authors declare that the research was conducted in the absence of any commercial or financial relationships that could be construed as a potential conflict of interest.

## Publisher’s Note

All claims expressed in this article are solely those of the authors and do not necessarily represent those of their affiliated organizations, or those of the publisher, the editors and the reviewers. Any product that may be evaluated in this article, or claim that may be made by its manufacturer, is not guaranteed or endorsed by the publisher.
