# Corrigendum: Anti-HIV-1 nanobody-IgG1 constructs with improved neutralization potency and the ability to mediate Fc effector functions

**DOI:** 10.3389/fimmu.2022.1091668

**Published:** 2022-11-22

**Authors:** Angela I. Schriek, Marlies M. van Haaren, Meliawati Poniman, Gillian Dekkers, Arthur E. H. Bentlage, Marloes Grobben, Gestur Vidarsson, Rogier W. Sanders, Theo Verrips, Teunis B. H. Geijtenbeek, Raimond Heukers, Neeltje A. Kootstra, Steven W. de Taeye, Marit J. van Gils

**Affiliations:** ^1^ Department of Medical Microbiology, Amsterdam UMC, Amsterdam Institute for Infection and Immunity, University of Amsterdam, Amsterdam, Netherlands; ^2^ QVQ Holding BV, Utrecht, Netherlands; ^3^ Department of Experimental Immunohematology, Sanquin Research and Landsteiner Laboratory, Amsterdam UMC, University of Amsterdam, Amsterdam, Netherlands; ^4^ Department of Microbiology and Immunology, Weill Medical College of Cornell University, New York, NY, United States; ^5^ Department of Biology, Faculty of Sciences, Utrecht University, Utrecht, Netherlands; ^6^ VerLin BV, Utrecht, Netherlands; ^7^ Department of Experimental Immunology, Amsterdam UMC, Amsterdam Institute for Infection and Immunity, University of Amsterdam, Amsterdam, Netherlands

**Keywords:** HIV-1, nanobodies, neutralization, Fc fusion, Fc-mediated effector functions

In the published article, there was an error in the Funding statement. The funder was mentioned as AIDSfonds and Aids fonds instead of Aidsfonds. The correct Funding statement appears below.

## Funding

This work was supported by HealthHolland/Aidsfonds: LSHM19101/P-44802, by HealthHolland/AMC: 2019-1167 and the AMC Fellowship from Amsterdam UMC received by MJG. ST is supported by Young investigator grant P-53301 Aidsfonds.

The authors apologize for this error and state that this does not change the scientific conclusions of the article in any way. The original article has been updated.

## Publisher’s note

All claims expressed in this article are solely those of the authors and do not necessarily represent those of their affiliated organizations, or those of the publisher, the editors and the reviewers. Any product that may be evaluated in this article, or claim that may be made by its manufacturer, is not guaranteed or endorsed by the publisher.

