# Supplementary material for: Anti-HIV-1 Nanobody-IgG1 Constructs With Improved Neutralization Potency and the Ability to Mediate Fc Effector Functions
Source: Front Immunol. 2022 May 16;13:893648. doi: 10.3389/fimmu.2022.893648 (PMC9150821; doi:10.3389/fimmu.2022.893648)
Supplement: Supplementary file 1 [file DataSheet_1.pdf]

	Subtype	J3	J3-J3	J3-J3-J3	2E7	2E7-2E7	1F10	1F10-1F10
**25710-2.43**	C	589	769	960	165	608	Nb	205
**Ce1176_A3**	C	547	726	940	123	505	59	398
**CH119.10**	CRF01_BC	578	697	866	122	483	151	186
**CNE55**	AE	505	702	905	68	374	Nb	59
**BJOX002000.03.2**	CRF01_BC	581	632	874	216	346	Nb	Nb
**Ce7030**	CRF01_AE	273	214	567	104	178	Nb	Nb
**246-F3_C10**	AC	711	779	827	117	254	Nb	58

Supplementary Figure 1. The average AUC of OD_{450} against the trimeric SOSIP Envs as determined by ELISA. Nb indicates no binding determined. Data shown are the average of three independent experiments.

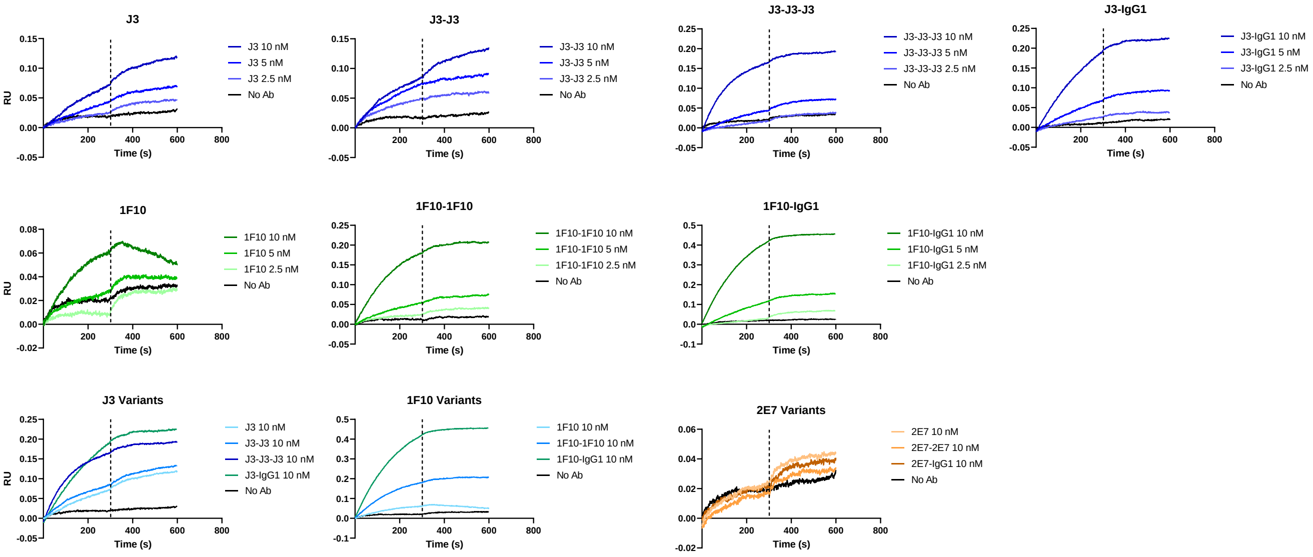

Supplementary Figure 2. Characterization of antibody-antigen interactions using biolayer interferometry. Biotinylated BG505 SOSIP.664 was loaded on streptavidin sensors, washed (baseline) and allowed to bind the antibody (association, indicated with a dotted line). The sensors (loaded with the antibody-antigen complex) are then dipped in running buffer to measure dissociation. Graphs visualize this for J3 and 1F10 nanobodies and nanobody-IgG1 fusions. Kd values were determined using Octet System Data Analysis Software.

	Subtype	J3	J3-J3	J3-J3-J3	J3-IgG1	J3-2E7	J3-2E7-IgG1	J3-1F10	J3-1F10-IgG1
**398F1**	A	33	9	12	3	57	11	9	6
**TRO.11**	B	74	35	47	44	48	70	86	52
**X2278**	B	66	172	48	27	19	51	50	51
**25710-2.43**	C	2	2	1	2	2	4	7	4
**Ce1176_A3**	C	9	6	4	3	7	13	16	15
**CNE8**	C	101	64	40	18	4	86	181	160
**CNE55**	AE	147	51	29	23	20	35	147	45
**Ce703010217_B6**	CRF01_AE	114	99	50	47	22	62	253	132
**246-F3_C10_2**	AC	19	8	8	1	1	3	10	11
**BJOX002000.03.2**	CRF01_BC	1	0,4	0,5	0,3	0,5	7	1	3
**CH119.10**	CRF01_BC	7	3	3	3	5	10	16	21
**X1632-S2-B10**	G	131	16	14	7	30	34	63	25

Supplementary Figure 3. Midpoint neutralization concentrations (IC_{50}) of J3, J3-2E7 and J3-1F10 nanobody and nanobody-IgG1 variants against HIV-1 pseudoviruses from the global panel in TZM-bl cells. Data shown are the average of three independent experiments (represented in nM).

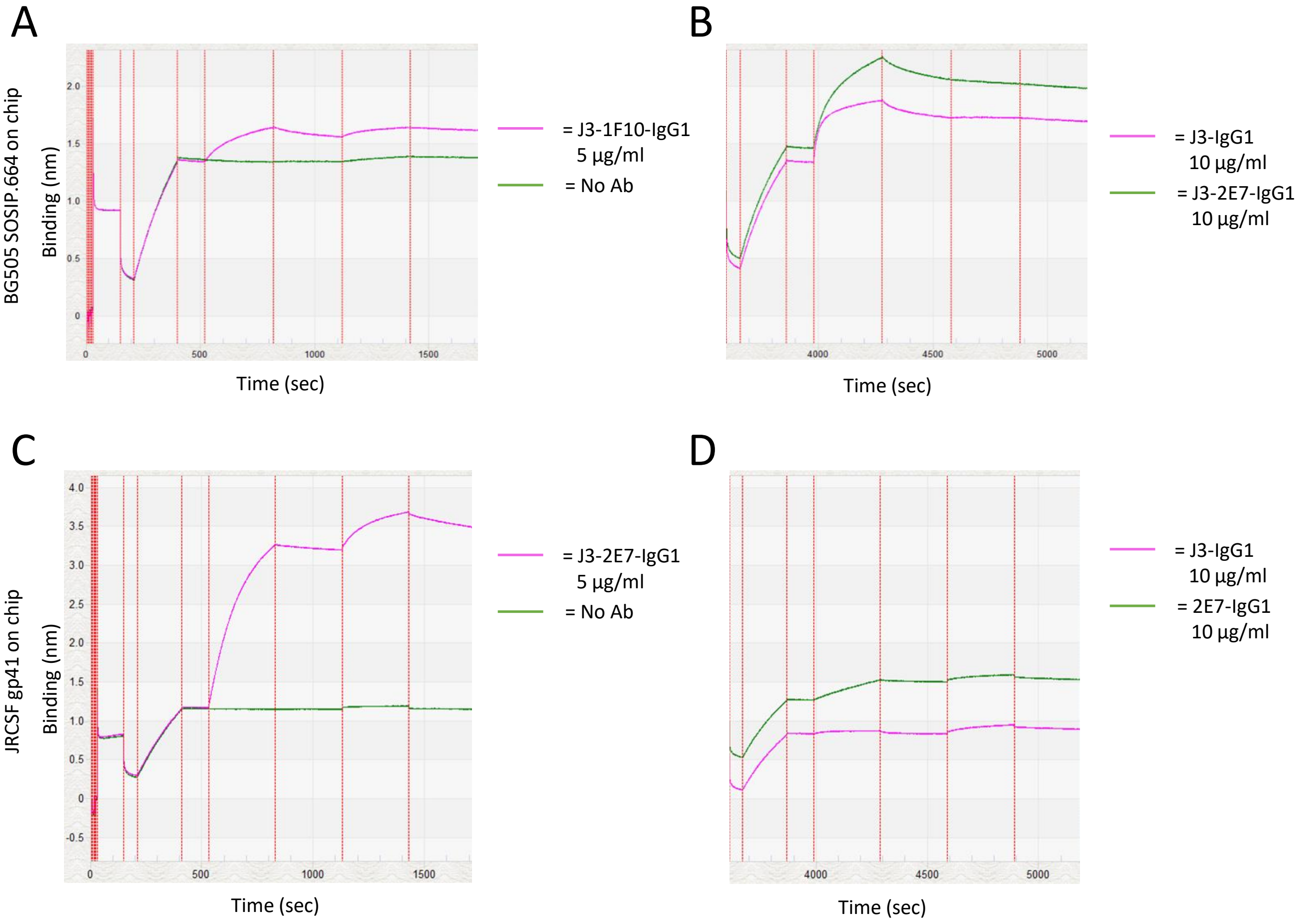

Supplementary Figure 4. Octet to confirm bispecificity of the bispecific nanobody-IgGs. A-B: Protein loaded on the chip was BG505 SOSIP.664 (binder: J3) and second protein 93IN905 gp120 (binder: 1F10). (A) J3-1F10-IgG1 and no antibody control. (B) J3-IgG1 and J3-2E7-IgG1. C-D: Protein loaded on the chip was JRCSF gp41 (binder: 2E7) and second protein BG505 gp120 (binder: J3). (C) J3-2E7-IgG1 and no antibody control. (D) J3-IgG1 and 2E7-IgG1 .

	Subtype	1F10	1F10-1F10	1F10-IgG1	2E7	2E7-2E7	2E7-IgG1
**398F1**	A	0,81	0,78	1,40	1,28	1,20	0,94
**TRO.11**	B	>1	>1	>1	2,10	0,93	1,28
**X2278**	B	0,20	0,08	0,24	0,46	0,39	1,32
**25710-2.43**	C	0,09	0,08	0,13	1,45	1,07	>1,5
**Ce1176_A3**	C	0,48	0,29	0,58	0,98	0,87	0,49
**CNE8**	C	0,22	0,23	0,09	>1,5	>1,5	1,39
**CNE55**	AE	>1	>1	>1	>1,5	1,42	1,13
**Ce703010217_B6**	CRF01_AE	0,63	0,42	1,29	2,24	2,15	1,17
**246-F3_C10_2**	AC	0,25	0,24	0,67	1,65	1,51	1,37
**BJOX002000.03.2**	CRF01_BC	0,09	0,09	0,14	1,11	0,43	1,09
**CH119.10**	CRF01_BC	0,85	0,56	1,027	1,45	1,23	1,41
**X1632-S2-B10**	G	1,99	0,18	0,62	1,52	0,98	1,22

Supplementary Figure 5. Midpoint neutralization concentrations (IC_{50}) of 1F10 and 2E7 variants against HIV-1 pseudoviruses from the global panel in TZM-bl cells. Non neutralizing is depicted as >1 or >1.5 (μM). Data shown are the average of three independent experiments (represented in μM).

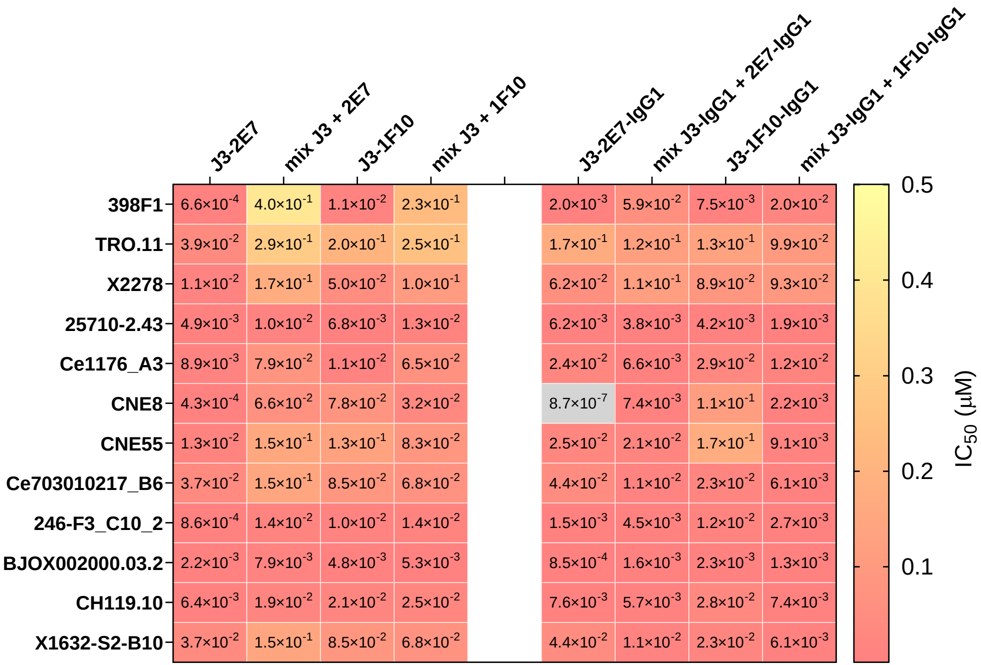

Supplementary Figure 6. Midpoint neutralization concentrations (IC_{50}) of bispecific nanobodies and nanobody-IgG1s compared to a mixture of corresponding nanobodies or nanobody-IgG1s against 12 viruses from the global panel. Data shown is the results of one experiment in duplicate.

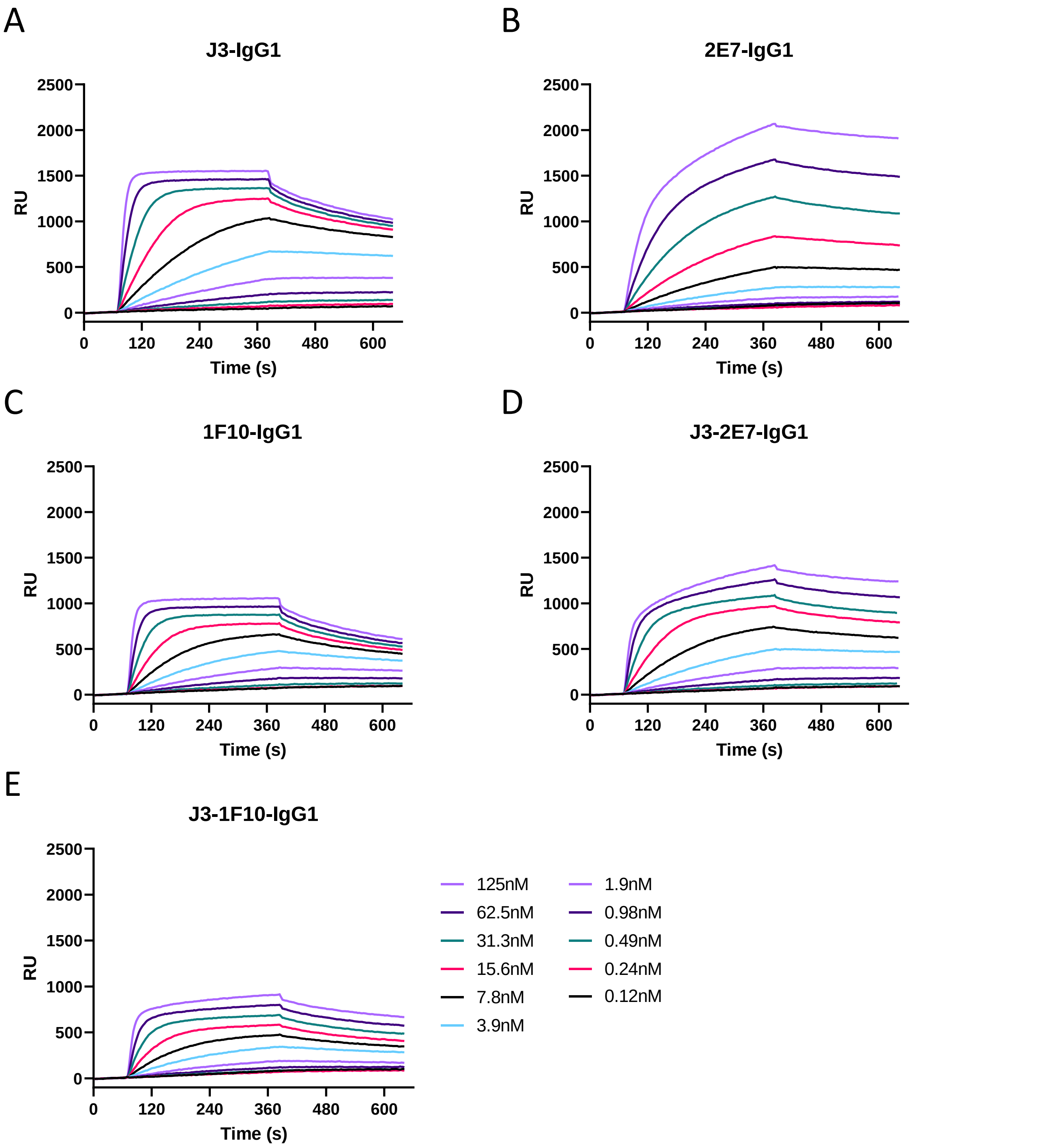

Supplementary Figure 7. Surface plasmon resonance results of the interaction between different nanobody-IgG molecules and hFcRn, recorded at pH 6.0. Power fitting at Rmax=1000RU was used.

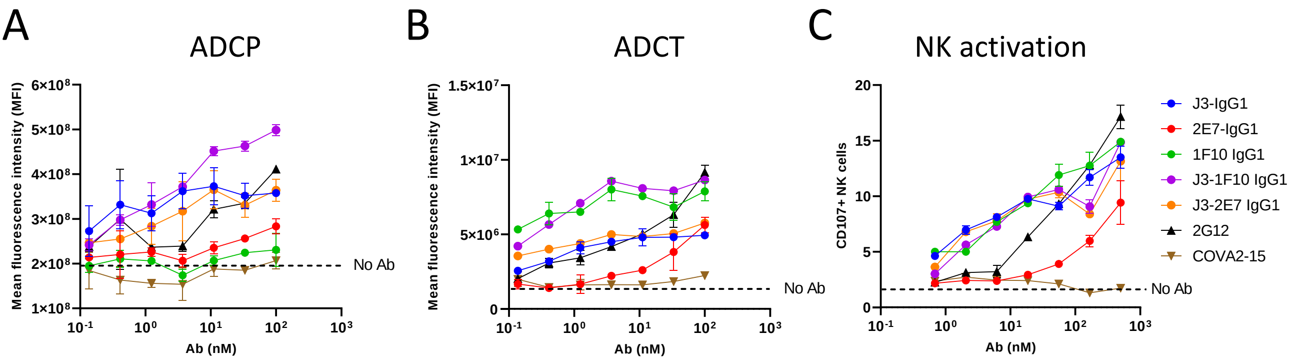

Supplementary Figure 8. Representative dose response curves of Fc-mediated effector functions. (A) Antibody-dependent cellular phagocytosis measured by the uptake of CNE55 SOSIP.v9.0 coated beads by THP-1 cells. (B) Antibody-dependent cellular trogocytosis measured by the uptake of membrane fragments from PHK26 stained BG505 gp160 expressing HEK cells by THP-1 cells and (C) NK activation after binding to Ce1176 SOSIP.v9.0 measured by the upregulation of CD107.
